# Dysregulated Gene Expression in the Primary Osteoblasts and Osteocytes Isolated from Hypophosphatemic *Hyp* Mice

**DOI:** 10.1371/journal.pone.0093840

**Published:** 2014-04-07

**Authors:** Kazuaki Miyagawa, Miwa Yamazaki, Masanobu Kawai, Jin Nishino, Takao Koshimizu, Yasuhisa Ohata, Kanako Tachikawa, Yuko Mikuni-Takagaki, Mikihiko Kogo, Keiichi Ozono, Toshimi Michigami

**Affiliations:** 1 Department of Bone and Mineral Research, Osaka Medical Center and Research Institute for Maternal and Child Health, Izumi, Osaka, Japan; 2 Department of Pediatrics, Osaka University Graduate School of Medicine, Suita, Osaka, Japan; 3 The First Department of Oral and Maxillofacial Surgery, Osaka University Graduate School of Dentistry, Suita, Osaka, Japan; 4 Department of Molecular and Cellular Biology of Mineralized Tissues, Kanagawa Dental College, Yokosuka, Kanagawa, Japan; Nihon University School of Medicine, Japan

## Abstract

Osteocytes express multiple genes involved in mineral metabolism including PHEX, FGF23, DMP1 and FAM20C. In *Hyp* mice, a murine model for X-linked hypophosphatemia (XLH), Phex deficiency results in the overproduction of FGF23 in osteocytes, which leads to hypophosphatemia and impaired vitamin D metabolism. In this study, to further clarify the abnormality in osteocytes of *Hyp* mice, we obtained detailed gene expression profiles in osteoblasts and osteocytes isolated from the long bones of 20-week-old *Hyp* mice and wild-type (WT) control mice. The expression of *Fgf23*, *Dmp1*, and *Fam20c* was higher in osteocytic cells than in osteoblastic cells in both genotypes, and was up-regulated in *Hyp* cells. Interestingly, the up-regulation of these genes in *Hyp* bones began before birth. On the other hand, the expression of *Slc20a1* encoding the sodium/phosphate (Na^+^/Pi) co-transporter Pit1 was increased in osteoblasts and osteocytes from adult *Hyp* mice, but not in *Hyp* fetal bones. The direct effects of extracellular Pi and 1,25-dihydroxyvitamin D_3_ [1,25(OH)_2_D_3_] on isolated osteoblastic and osteocytic cells were also investigated. Twenty-four-hour treatment with 10^−8^ M 1,25(OH)_2_D_3_ increased the expression of *Fgf23* in WT osteoblastic cells but not in osteocytic cells. *Dmp1* expression in osteocytic cells was increased due to the 24-hour treatment with 10 mM Pi and was suppressed by 10^−8^ M 1,25(OH)_2_D_3_ in WT osteocytic cells. We also found the up-regulation of the genes for FGF1, FGF2, their receptors, and Egr-1 which is a target of FGF signaling, in *Hyp* osteocytic cells, suggesting the activation of FGF/FGFR signaling. These results implicate the complex gene dysregulation in osteoblasts and osteocytes of *Hyp* mice, which might contribute to the pathogenesis.

## Introduction

Osteocytes are dendritic cells that differentiate from osteoblasts and are embedded within the bone matrix [1]. Although their location and inaccessibility in the mineralized bone matrix delayed the analyses of their function at cellular and molecular levels, recent studies have uncovered the profound roles of osteocytes in bone homeostasis. Osteocytes were shown to be responsible for sensing mechanical strain and controlling bone formation and resorption [2], [3]. Mature osteocytes produce sclerostin, which binds to low density lipoprotein receptor-related protein 5 and 6 (Lrp5/6) to inhibit Wnt/β-catenin signaling [4]. Mechanical strain as well as parathyroid hormone (PTH) reduces the expression of sclerostin in osteocytes, which in turn enhances Wnt/β-catenin signaling and increases bone formation [5], [6]. Moreover, receptor activator of nuclear factor kappa B ligand (RANKL) produced by osteocytes was shown to play a central role in the control of bone mass by specifically deleting its expression in osteocytes [7], [8].

In addition to these important roles in bone homeostasis, accumulating evidence has indicated that osteocytes play a key role in mineral metabolism. Fibroblast growth factor 23 (FGF23), which is a circulating factor that regulates the renal reabsorption of phosphate (Pi) and metabolism of vitamin D, has been shown to be mainly produced by osteocytes [9], [10]. FGF23 increases renal Pi excretion by reducing the expression of type IIa and IIc sodium/phosphate (Na^+^/Pi) co-transporters (NPTIIa and NPTIIc) and decreases the production of 1,25-dihydroxyvitamin D [1,25(OH)_2_D] in the renal proximal tubules [11], [12], [13]. Activating mutations in the gene for FGF23 cause autosomal dominant hypophosphatemic rickets (ADHR; OMIM #193100), which is characterized by renal Pi wasting, hypophosphatamia, and an inappropriately low level of serum 1,25(OH)_2_D [14].

In addition to FGF23, several other molecules involved in Pi homeostasis are also highly expressed in osteocytes. Among them, the *phosphate-regulating gene homologous to endopeptidase on X chromosome (PHEX)*, whose product is a member of the M13 family of type II cell-surface zinc-dependent proteases, is responsible for X-linked hypophosphatemia (XLH; OMIM#307800), the most common form of hereditary hypophosphatemic rickets [15], [16]. *Dentin matrix protein 1 (DMP1)*, which encodes an extracellular matrix protein belonging to the SIBLING (small integrin-binding ligand, N-linked glycoproteins) family, is also mainly expressed in osteocytes and is responsible for autosomal recessive hypophosphatemic rickets type I (ARHR1; OMIM#241520) [17], [18]. Another molecule named Fam20c (family with sequence similarity 20c), which is highly expressed in mineralized tissues including osteocytes, was also recently suggested to be involved in Pi homeostasis [19]. Fam20c has been identified as a secreted kinase that phosphorylates the extracellular proteins involved in biomineralization, including members of the SIBLING family such as DMP1, osteopontin, and MEPE (matrix extracelular phosphoglycoprotein) [20]. *Fam20c*-knockout mice were reported to exhibit hypophosphatemic rickets [19]. In humans, FAM20C is known to be responsible for Raine syndrome, which is characterized by osteosclerosis and ectopic calcification, and an exome sequencing study has recently revealed the association of mutations in this gene with hypophosphatemia [21]. Inactivation of the genes *Phex*, *Dmp1*, or *Fam20c* was shown to result in the increased expression of *Fgf23* in osteocytes [17], [18], [19], [22], [23], which suggests that these molecules negatively regulate the expression of *Fgf23*. Thus, these *in vivo* findings have indicated the critical roles of the intimate relationships among these molecules in osteocytes in the regulation of mineral metabolism.

Hypophosphatemic *Hyp* mouse carries a large 3′-deletion in the *Phex* gene and is widely used as a model for human XLH [24], [25]. In the current study, to investigate in detail the abnormality in osteoblasts and osteocytes of *Hyp* mice, we isolated cells of the osteoblast/osteocyte lineage from *Hyp* and wild-type mice based on the differentiation stage, and obtained detailed gene expression profiles. Interestingly, the expression of *Dmp1, Fam20c,* and *Slc20a1* encoding type III Na^+^/Pi co-transporter Pit1 was found to be markedly elevated in *Hyp* cells. The increased expression of *Dmp1* and *Fam20c* in *Hyp* bones was observed even before birth, as was that of *Fgf23*. The expression of the genes for multiple FGFs and their receptors was up-regulated in *Hyp* osteocytic cells, suggesting the activated FGF/FGFR signaling. The effects of extracellular Pi and active vitamin D on gene expression were also analyzed using isolated osteoblastic and osteocytic cells.

## Materials and Methods

### Animals

The animal experiments were carried out in accordance with the “Guidelines for Proper Conduct of Animal Experiments” formulated by the Scientific Council of Japan. The protocols were approved by the Institutional Animal Care and Use Committee of the Osaka Medical Center and Research Institute for Maternal and Child Health (Permit number: BMR24–1). All mice used in the study were of the C57BL/6J strain. Wild-type (WT) mice were obtained from Clea Japan Inc. (Tokyo, Japan). Hypophosphatemic *Hyp* mice were initially obtained from the Jackson Laboratory (Bar Harbor, ME), and male hemizygotes (*Phex^Hyp^/Y*) and female heterozygotes (*Phex^Hyp/+^*) were produced for use in experiments. *Hyp* genotyping was performed by genomic PCR using the following primer set: *Phex* forward primer, 5′-CCAAAATTGTTCTTCAGTACACC-3′, and *Phex* reverse primer, 5′-ATCTGGCAGCACACTGGTATG-3′ [26]. A 258-bp PCR product was obtained from the WT *Phex* allele, but not from the *Hyp* allele. Mice were maintained in a pathogen-free barrier facility, fed standard mouse chow ad libitum, and exposed to a 12-h light, 12-h dark cycle.

### Assay for Biochemical Parameters

Plasma Pi levels were determined using a commercial kit (Wako, Osaka, Japan). Intact FGF23 levels were assayed using a sandwich ELISA kit (Kainos Laboratories, Inc., Tokyo, Japan).

### Isolation of Primary Osteoblast/Osteocyte Lineage Cells from Mouse Long Bones

Primary osteoblasts and osteocytes were isolated from mouse long bones, according to a method previously described by Mikuni-Takagaki, et al. with some modifications [27]. Briefly, mouse tibiae and femurs were minced into 0.5-mm pieces and digested with 1.25 mg/ml collagenase (Wako, Osaka, Japan) in Ca^2+^-free, Mg^2+^-free Hanks’ Balanced Salt Solution (HBSS) at 37°C. Cells released after the first and second (15 min. each) and third to fifth (20 min. each) digestion were collected through a 100-μm nylon cell strainer as Fractions 1 to 5, respectively. Residual bone pieces were treated with 4 mM EGTA in Ca^2+^-free, Mg^2+^-free HBSS for 15 min. and then with 1.25 mg/ml collagenase for 20 min. at 37°C to collect the cells as Fractions 6 and 7, respectively. Bone pieces were subsequently treated with 4 mM EGTA for 15 min. and then with 1.25 mg/ml collagenase for 20 min. to collect cells as Fractions 8 and 9. Based on the gene expression of freshly isolated cells, Fractions 6–9 were considered to be osteocyte-rich fractions.

### Collagen-gel Embedded Culture of Primary Osteoblasts and Osteocytes

To examine the direct effects of 1,25(OH)_2_D and Pi on primary osteoblastic and osteocytic cells, a collagen-embedded culture was performed using acid-soluble type I collagen (3 mg/ml) derived from porcine tendon (Cellmatrix Type I-A; Nitta Gelatin, Osaka, Japan). To make a collagen solution, Cellmatrix Type I-A, 10-fold concentrated αMEM, and reconstruction buffer (260 mM NaHCO_3_, 200 mM HEPES, and 50 mM NaOH) were mixed at a proportion of 8∶1∶1 by volume at 4°C. Osteocytic cells (Fractions 6–9) or osteoblastic cells (Fractions 3–5) isolated from mouse long bones as described above were suspended in the collagen solution at the density of 2×10^7^ cells/ml. The cell suspension was kept on ice to prevent gel formation. Fifty μl of gel solution with the cells was then gently dropped onto a well in a 24-well culture plate, and gelled immediately by keeping the plate at 37°C for 15 min. After the gel formed, alpha MEM supplemented with 5% fetal bovine serum (FBS), 5% calf serum (CS), 100 units/ml penicillin, and 100 μg/ml streptomycin containing stimulants or vehicle was overlayed, and the cells were cultured at 37°C in a 5% CO_2_ atmosphere until analysis.

### RNA Extraction and Real-time Polymerase Chain Reaction (PCR) Analysis

Total RNA was extracted using TRIzol Reagent (Invitrogen, Carlsbad, CA), and then treated with DNase (Qiagen). DNase-treated total RNA was reverse transcribed using random hexamers (Promega, Madison, WI) and SuperScript II (Invitrogen). We utilized TaqMan Gene Expression Assays with the 7300 Real Time PCR System (Applied Biosystems) for real-time PCR. To generate a standard curve for real-time PCR, the amplicons of interest were first cloned into a pT7-Blue vector (Novagen, Madison, WI), and serial 10-fold dilutions of the constructed plasmid were included in the assay. The copy number of the target cDNA in each sample was estimated by referring to the standard curve, which was standardized to that of *Gapdh* in each sample.

### Immunohistochemistry

Tibiae obtained from 20-week-old mice were fixed in 4% paraformaldehyde solution, undecalcified in 500 mM EDTA, and embedded in paraffin. Sections were deparaffinized, rehydrated, and subjected to quenching with endogenous peroxidase, and immunostained using an anti-DMP1 rabbit polyclonal antibody (Takara, Shiga, Japan) and the ImmunoCruz Staining System (Santa Cruz Biotechnology, Santa Cruz, CA). Normal IgG was used as a negative control. Sections were counterstained with hematoxylin.

### Statistical Analysis

Data from the gene expression profiles in the cells of each fraction were analyzed using Kruskal-Wallis one-way analysis, and the significance in the differences among the groups was assessed by the Shirley-Williams test. We used the Mann-Whitney’s U test to compare the data between 2 groups. To compare the data among more than 3 groups, we used the one-way analysis of variance (ANOVA) with Tukey’s post hoc test.

## Results

### Gene Expression Profile in Osteoblasts and Osteocytes Freshly Isolated from Mouse Long Bones

**Figure 1:**
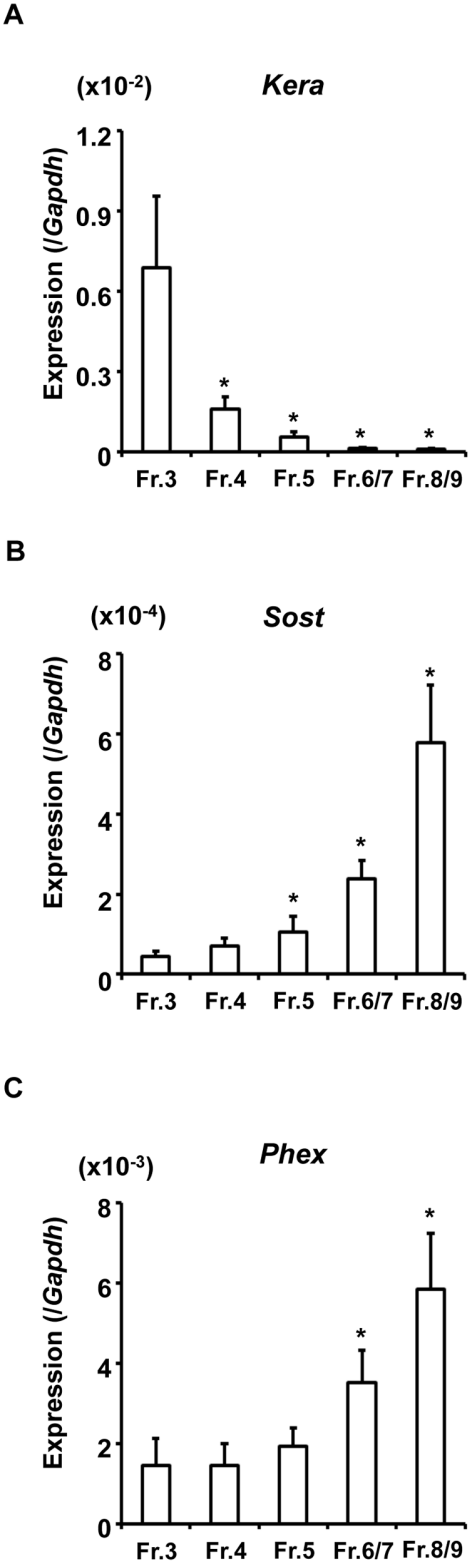
Expression of the marker genes for osteoblasts and osteocytes in cells isolated from WT mice. Long bones from 20-week-old male WT mice were minced, and were subjected to sequential treatment with collagenase and EGTA, and cells were collected as a fraction after each treatment. Fractions 6 and 7 as well as Fractions 8 and 9 were combined because of low cell numbers. RNA extracted from fresh cells in Fractions 3 to 8/9 was subjected to real-time PCR to examine the expression of *Kera* (A), *Sost* (B), and *Phex* (C). The copy number of the target cDNA in each sample was estimated by referring to a standard curve, which was standardized to that of *Gapdh*. Data are shown as the mean ± SEM of 4 isolations. In each isolation procedure, 4 mice were used. *p<0.05 vs. Fr. 3.

To confirm the successful isolation of osteoblasts and osteocytes from mouse long bones, we analyzed gene expression in fresh cells isolated from WT mice (C57BL/6J, male, 20-week-old). Minced long bones were subjected to sequential treatment with collagenase and EGTA as described in the Materials and Methods section, and cells were collected as a fraction after each treatment. Fractions 6 and 7 were combined (described as Fraction 6/7) as well as Fractions 8 and 9 (described as Fraction 8/9) because of the lower number of cells. Fractions 1 and 2 were discarded because these fractions contained abundant fibroblasts derived from the soft tissue surrounding bones. We performed real-time PCR analyses using RNA extracted from fresh cells in each fraction of Fractions 3 to 8/9 to examine the expression of osteoblast and osteocyte marker genes. The expression of *Kera*, the gene encoding the proteoglycan keratocan, was previously reported to be higher in osteoblasts *in vivo* [28]. In our isolation, the expression of *Kera* was the strongest in Fraction 3 and was almost undetectable in Fractions 6/7 and 8/9. On the other hand, the expression of *Sost*, a marker for mature osteocytes, was higher in Fractions 6/7 and 8/9 than in Fraction 3, and was the highest in Fraction 8/9 (Fig. 1). These results confirmed that cells of the osteoblast/osteocyte lineage were sequentially isolated based on their differentiation stages, and we considered that Fractions 3–5 were osteoblast-rich while Fractions 6/7 and 8/9 were osteocyte-rich. We also examined the expression of *Phex*, and found that it was higher in the later fractions, which suggested the dominant expression of *Phex* in osteocytes (Fig. 1).

### Increased Expression of *Fgf23*, *Dmp1*, *Fam20c*, and *Slc20a1* in *Hyp* Cells of the Osteoblast/Osteocyte Lineage

**Figure 2:**
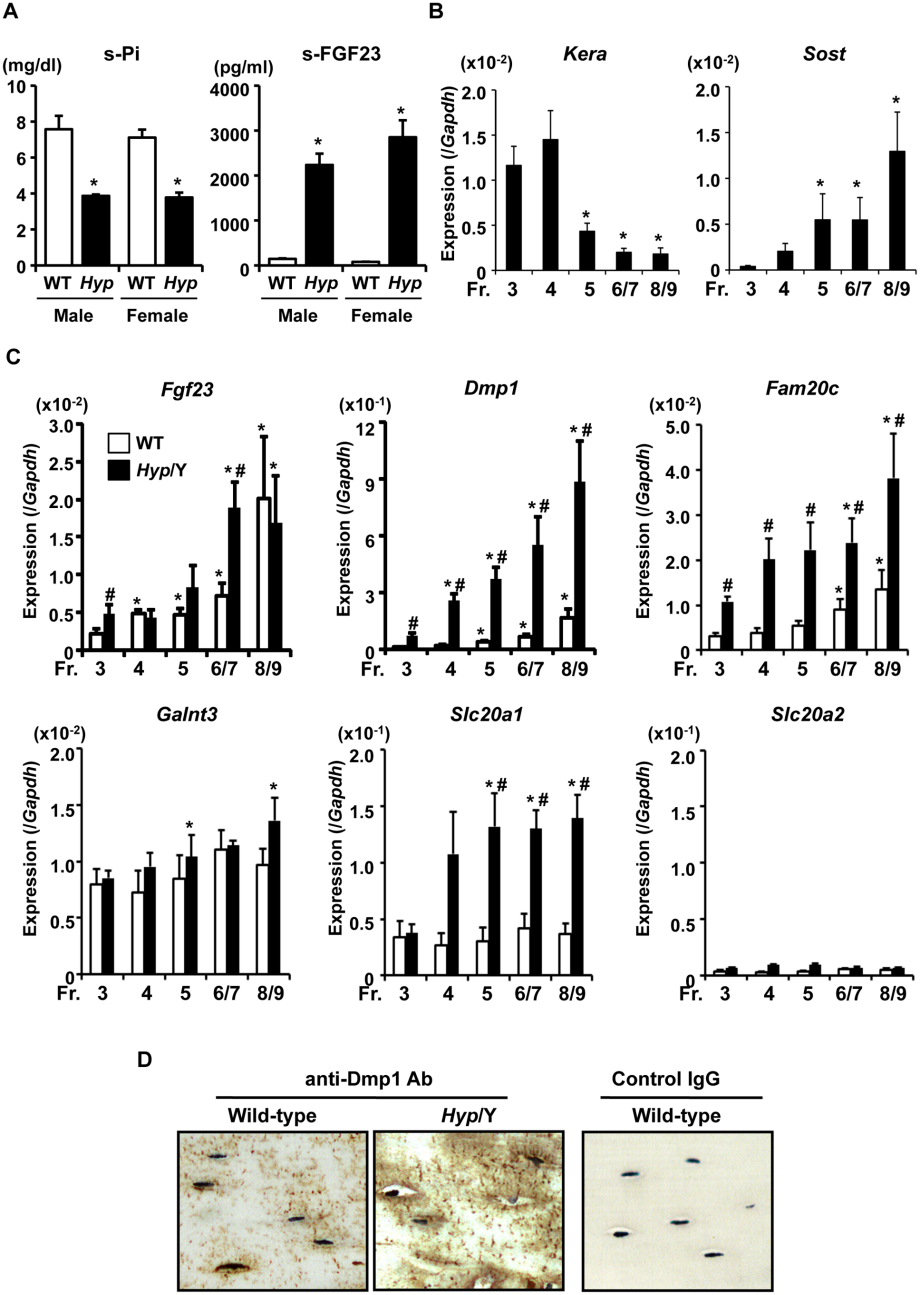
Altered gene expression in osteoblasts and osteocytes isolated from *Hyp* mice. (**A**) Serum Pi and FGF23 levels in 20-week-old *Hyp* (male hemizygotes and female heterozygotes, black bars) and WT (white bars) mice. Data are shown as the mean ± SEM (n = 3–4). *p<0.05 vs. WT. (**B**) Expression of the osteoblastic marker *Kera* and osteocytic marker *Sost* in fresh cells isolated from the long bones of 20-week-old male *Hyp* mice. Real-time PCR was performed, and expression of the target cDNA was standardized to that of *Gapdh*. Data are shown as the mean ± SEM of 4 isolations. In each isolation procedure, 4 mice were used. *p<0.05 vs. Fr. 3. (**C**) Differential expression of the genes involved in Pi metabolism in osteoblasts and osteocytes freshly isolated from the long bones of 20-week-old *Hyp* (black bars) or WT (white bars) male mice. Real-time PCR was performed to examine the expression of *Fgf23, Dmp1, Fam20c, Galnt3, Slc20a1*, and *Slc20a2*. Data are shown as the mean ± SEM of 4 (in WT) or 5 (in *Hyp*) isolations. In each isolation procedure, 4 mice were used. *p<0.05 vs. Fr. 3; ^#^ *p*<0.05 vs. WT. (**D**) Immunostaining of tibiae from WT and *Hyp* male mice with the anti-Dmp1 antibody. The nuclei of osteocytes were counterstained with hematoxylin.

We isolated osteoblasts and osteocytes from 20-week-old male WT and *Hyp* (*Phex^Hyp^/Y*) mice to compare the gene expression profiles between the genotypes. We confirmed hypophosphatemia and elevated levels of serum FGF23 in *Hyp* mice at this age in both male hemizygotes and female heterozygotes. No significant difference was observed in serum levels of Pi and FGF23 between male hemizygote and female heterozygote *Hyp* mice (Fig. 2A). DNase-treated total RNA samples were then prepared from fresh cells in Fractions 3, 4, 5, 6/7, and 8/9 for real-time PCR analyses. Similar to the case of WT, the expression of *Kera* was low while that of *Sost* was high in Fractions 6/7 and 8/9 isolated from *Hyp* bones, which suggested that these fractions were osteocyte-rich in both WT and *Hyp* (Fig. 1, 2B). Interestingly, the expression of *Sost* was higher in *Hyp* cells than in WT cells.

We then compared the expression of the genes involved in Pi metabolism in these fractions from *Hyp* and WT mice (Fig. 2C). The expressions of *Fgf23*, *Dmp1*, and *Fam20c* were all higher in the later fractions than in the earlier fractions in both genotypes, indicating the high expression of these genes in osteocytes. The expression of *Fgf23* was significantly higher in *Hyp* cells than in WT cells in Fractions 3 and 6/7, although this increase was not as high as expected from the elevated level in serum FGF23. The expression of *Dmp1* was markedly higher in *Hyp* cells than in WT cells through Fractions 3 to 8/9. Moreover, the expression of *Fam20c* was also higher in *Hyp* cells than in WT cells. Since the elevation in *Fgf23* expression in *Hyp* cells was modest, we examined the expression of *GalNAc transferase 3 (Galnt3)*, which encodes the enzyme involved in glycosylation of the FGF23 protein [29]. No significant difference was observed in the expression of *Galnt3* between the genotypes, although it was slightly increased in *Hyp* cells in Fractions 5 and 8/9 (Fig. 2C). We did not examine the expression of *Phex* in *Hyp* cells because of the large deletion in the gene.

The expression of type III Na^+^/Pi co-transporters was also analyzed. *Slc20a1* encoding Pit1 was highly expressed in osteoblasts and osteocytes, and its expression was increased in *Hyp* cells in Fractions 4 to 8/9. On the other hand, the expression of *Slc20a2* encoding Pit2 was low in all fractions (Fig. 2C). We also examined the expression of type IIa, IIb, and IIc Na^+^/Pi co-transporters, which was low in osteoblasts and osteocytes from both WT and *Hyp* mice (data not shown).

Immunohistochemical analysis was also performed to examine Dmp1 protein expression. Consistent with real-time PCR data, staining with the anti-Dmp1 antibody was more intense in *Hyp* bones (Fig. 2D).

### Increased Expression of *Fgf23, Dmp1,* and *Fam20c,* but not that of *Slc20a1* Occurred before Birth in *Hyp* Bones

**Figure 3:**
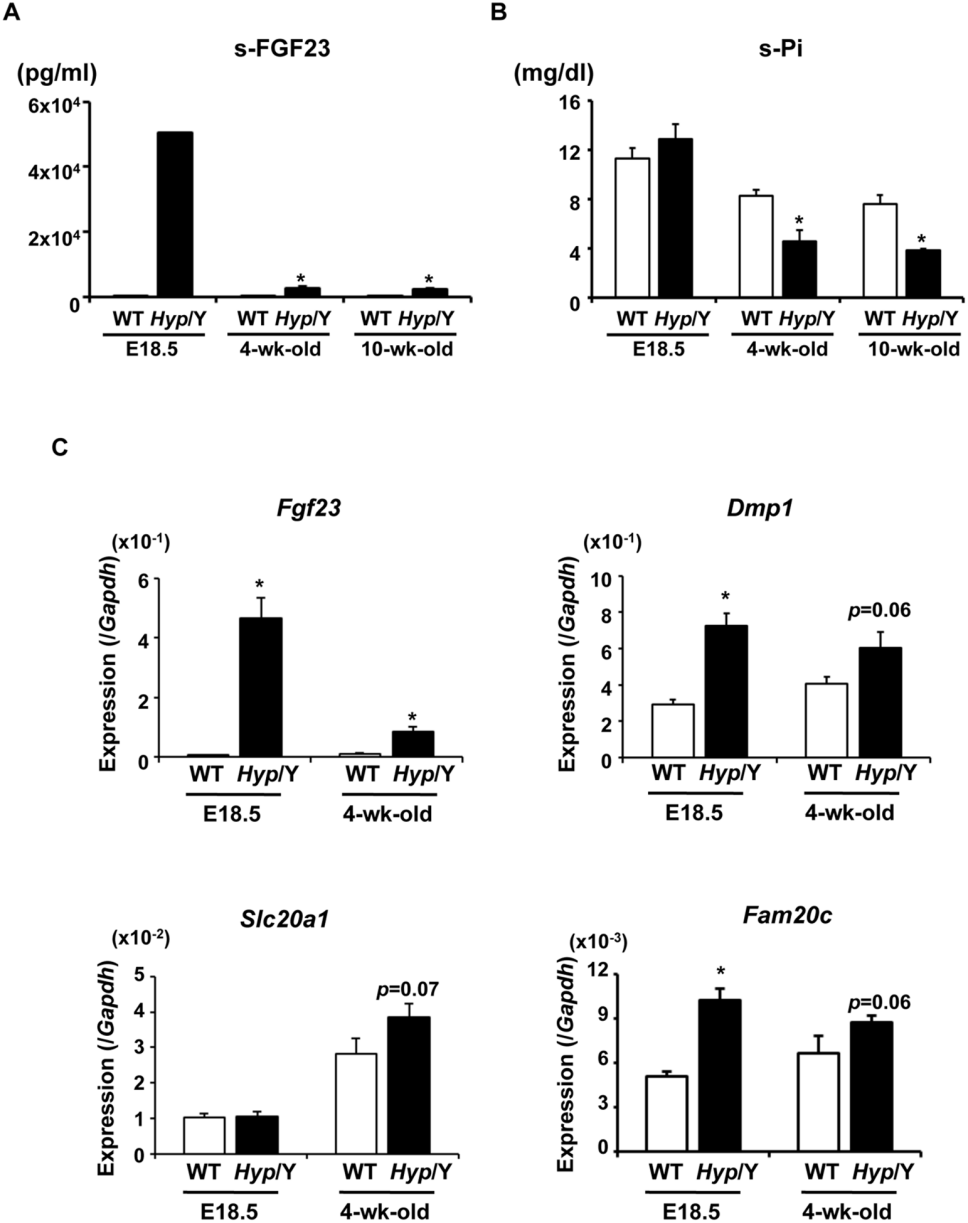
Increased expression of *Fgf23*, *Dmp1*, and *Fam20c* in *Hyp* bones occurred before birth. (**A**) Serum levels of FGF23 in WT (white bars) and *Hyp* (black bars) male mice at E18.5 and 4 and 10 weeks of age. Regarding samples at E18.5, serum from genetically identical littermates were pooled together and assayed as one sample. Regarding samples at 4 and 10 weeks of age, data are shown as the mean ± SEM (n = 3–4). *P<0.05 vs. WT. (**B**) Serum Pi levels in WT (white bars) and *Hyp* (black bars) male mice at E18.5 and 4 and 10 weeks of age. Data are shown as the mean ± SEM (n = 3–7). *P<0.05 vs. WT. (**C**) Gene expression in the bones of WT (white bars) and *Hyp* (black bars) male mice at E18.5 and 4 weeks of age. RNA was extracted from the bones after removal of the bone marrow and surrounding soft tissue, and was used for real-time PCR to examine the expression of the indicated genes. Expression of the target cDNA was standardized to that of *Gapdh*. Data are shown as the mean ± SEM. n = 5–9 in E18.5 fetuses. n = 3 in 4-week-old mice. *P<0.05 vs. WT.

To clarify whether the increased expression of *Fgf23*, *Dmp1*, *Fam20c* or *Slc20a1* occurred prenatally or after birth in *Hyp* bones, we analyzed gene expression in fetal bones. Female *Hyp* heterozygotes were mated with WT male mice, and their fetuses were obtained at E18.5. Genomic PCR for *Phex* and the male-specific gene *Sry* was performed to determine the *Phex* genotype and the gender, respectively, and male *Hyp* fetuses and WT littermates were used for analyses. Circulating levels of FGF23 in *Hyp* fetuses at E18.5 were markedly high, although plasma Pi levels were comparable between the genotypes (Fig. 3A, B). The expression of *Fgf23*, *Dmp1*, and *Fam20c* was higher in bones from *Hyp* fetuses than in those from WT fetuses (Fig. 3C), which suggests that the increase in the expression of these genes occurred before birth. On the other hand, the expression of *Slc20a1* was similar between the genotypes at E18.5, and appeared to slightly increase at the age of 4 weeks in *Hyp* (Fig. 3C). Based on this observation, we speculated that the elevated expression of *Slc20a1* observed in isolated osteoblasts and osteocytes from adult *Hyp* mice, which was shown in Figure 2, might be a compensation to adapt for the decrease in extracellular Pi levels after birth.

### Direct Effects of Extracellular Pi on Gene Expression in the Isolated Osteoblasts and Osteocytes of WT and *Hyp* Mice

**Figure 4:**
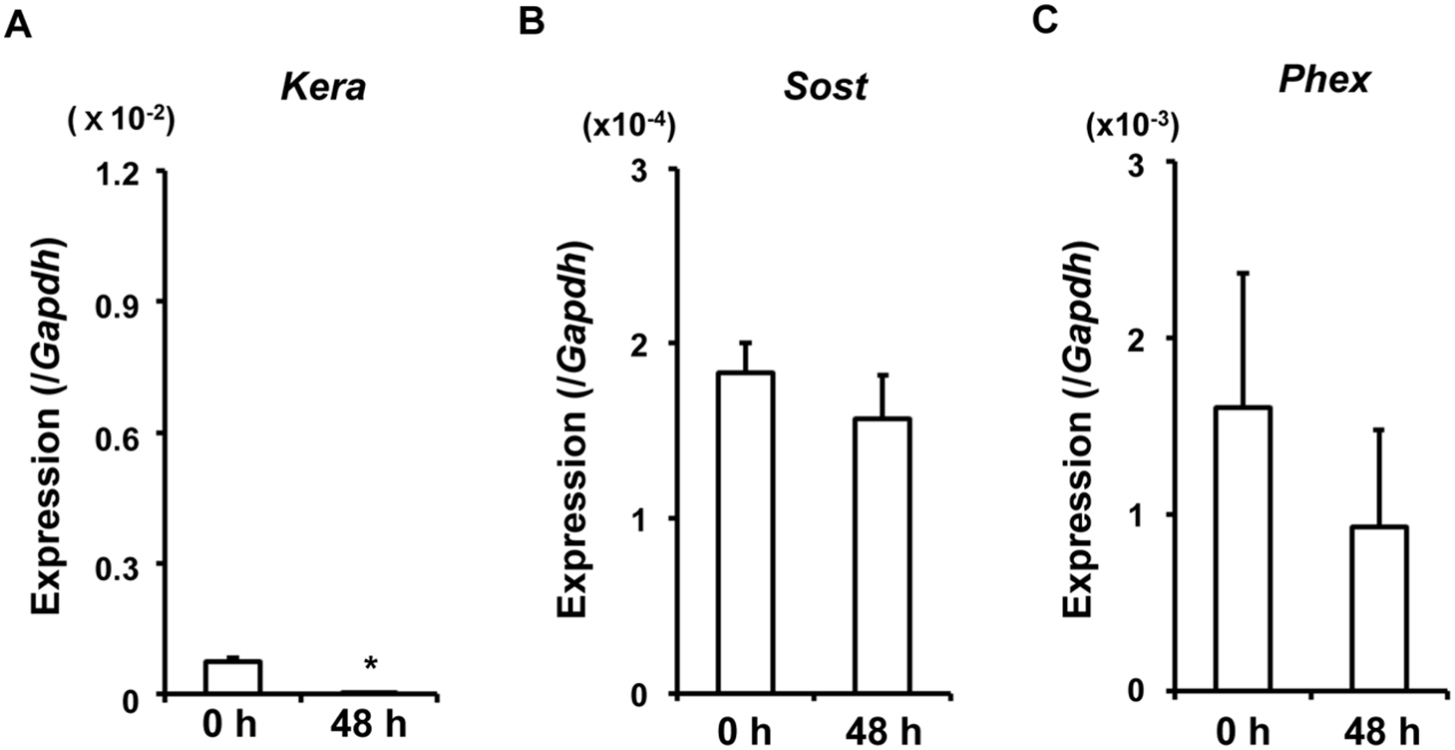
Expression of *Sost* and *Phex* was retained after 48-hour collagen-embedded culture of the isolated osteocytic cells. The fresh cells of osteocyte-rich Fractions 6–9 isolated from 10-week-old WT bones were aliquoted, and RNA was extracted immediately (0 h) or after 48-hour collagen-embedded culture (48 h). Real-time PCR was performed to examine the expression of the osteoblastic gene *Kera* (**A**) and osteocytic genes *Sost* (**B**) and *Phex* (**C**). The copy number of the target cDNA in each sample was estimated by referring to a standard curve, which was standardized to that of *Gapdh*. Data are shown as the mean ± SEM of 3 isolations. *p<0.05 vs. 0 h.

Next, we investigated the direct effects of extracellular Pi and 1,25(OH)_2_D_3_ on gene expression in the isolated primary osteocytic cells. For this purpose, we performed type I collagen-embedded culture as described in the Materials and Methods section. First, we examined whether the primary osteocytic cells retained the expression of osteocytic genes during the culture. Fresh cells of osteocyte-rich Fractions 6–9 isolated from 10-week-old WT bones were aliquoted, and RNA was extracted immediately or after 48-hour collagen-embedded culture for real-time PCR. There was no significant difference in the expression of *Sost* and *Phex* between the cells at isolation and those after 48-hour collagen-embedded culture (Fig. 4B, C). As to the expression of *Kera*, which is an osteoblastic marker gene, it remained low after the culture (Fig. 4A). These results suggested that the cells of Fractions 6–9 cultured in collagen gel retained the expression of osteocytic genes to some extent for 48 hours.

**Figure 5:**
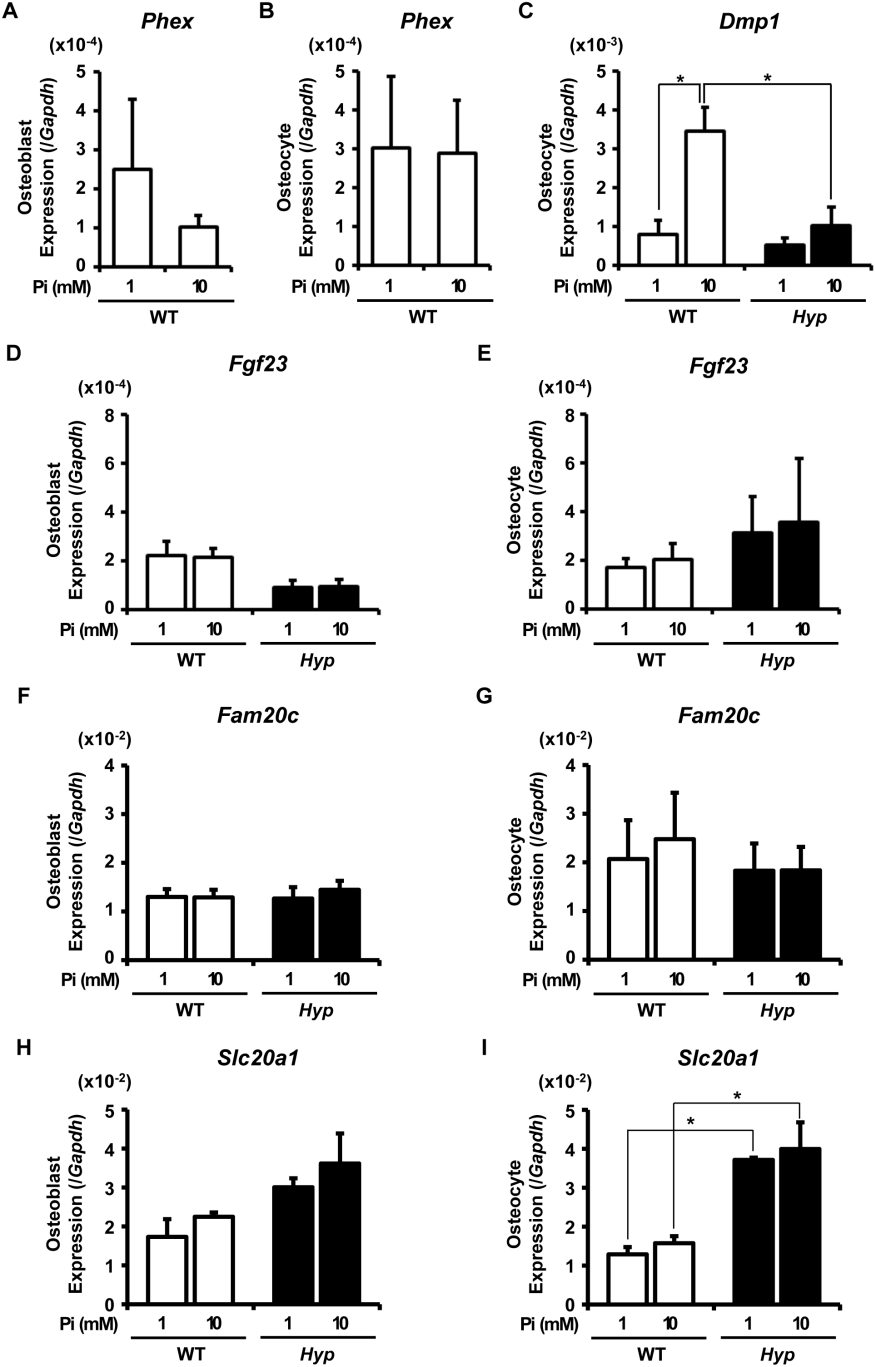
Direct effects of increased extracellular Pi on gene expression in WT and *Hyp* osteoblastic and osteocytic cells. The cells of osteoblast-rich Fractions 3–5 (**A, D, F, H**) or osteocyte-rich Fractions 6–9 (**B, C, E, G,** **I**) isolated from 10-week-old WT (white bars) and *Hyp* (black bars) bones were embedded in collagen gel and incubated in the presence of 1 mM or 10 mM Pi for 24 hours, after which RNA was extracted to analyze expression of the indicated genes by real-time PCR. Expression of the target cDNA in each sample was standardized to that of *Gapdh*. Data are shown as the mean ± SEM of 3 isolations. In each isolation procedure, 4 mice were used. *p<0.05.

Then, to examine the acute direct effects of extracellular Pi, the osteocytic Fractions 6–9 isolated from WT and *Hyp* bones were embedded in collagen gel and incubated in the presence of 1 mM or 10 mM Pi for 24 hours before RNA was extracted for analysis. For comparison, similar experiments were also performed using the osteoblastic Fractions 3–5 (Fig. 5). We used 10-week-old mice in these experiments, since the gene expression profiles in the freshly isolated cells from 10-week-old WT and *Hyp* mice was similar to those in the cells from 20-week-old mice shown in Fig. 2 (data not shown). Unexpectedly, although the expression of *Slc20a1* encoding Pit1 was still higher in *Hyp* osteocytic cells than in WT osteocytic cells after the 24-hour culture (Fig. 5I), the expressions of *Dmp1*, *Fgf23* and *Fam20c* in *Hyp* osteocytic cells were not higher than in WT cells after the culture, which was different from the case of the gene expression analyzed using the RNA extracted from the fresh cells at the isolation (Fig. 5C, E, G). Although the mechanism for this discrepancy remains unclear, the bone microenvironment appears to play a critical role in the regulation of these genes.

Among the genes analyzed, the expression of *Phex* in WT cells was not altered by the 24-hour treatment with increased Pi (Fig. 5A, B). Interestingly, an elevation in extracellular Pi resulted in a marked increase in *Dmp1* expression in WT osteocytic cells, while the up-regulation of *Dmp1* expression by the elevated Pi was not significant in *Hyp* cells (Fig. 5C). The expression of *Fgf23* and *Fam20c* was not obviously changed by the 24-hour treatment with increased extracellular Pi in either genotype (Fig. 5D–G). The expression of *Slc20a1* also was unaltered by the 24-hour treatment with high Pi in both WT and *Hyp* cells (Fig. 5H, I).

### Long-term Effects of Extracellular Pi on the *Slc20a1* Expression in Osteoblastic MC3T3-E1 Cells

**Figure 6:**
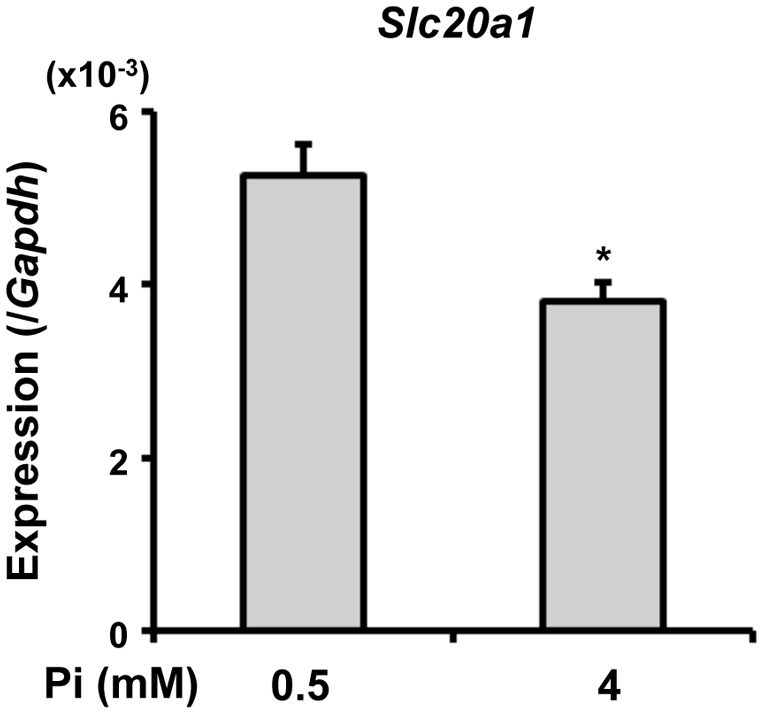
Long-term effects of extracellular Pi on the *Slc20a1* expression in osteoblastic MC3T3-E1 cells. MC3T3-E1 cells were plated in 6-well culture plates at a density of 1×10^3^ cells/well in alpha MEM supplemented with 10% FBS. From the next day, the cells were cultured in the presence of 0.5 mM or 4 mM Pi for 14 days. The media was prepared by adding sodium phosphate buffer to Pi-free media supplemented with 10% FBS. The concentration of sodium was adjusted by adding sodium sulfate buffer. After 14 days of culture, RNA was extracted to examine the expression of *Slc20a1* by real-time PCR. Data are shown as the mean ± SEM (n = 3). *p<0.05 vs. 0.5 mM.

As described above, the 24-hour treatment with high extracellular Pi did not alter the expression of *Slc20a1* in primary osteoblasts and osteocytes from either genotype (Fig. 5H, I). However, since the *Slc20a1* expression was up-regulated in the osteoblasts and osteocytes freshly isolated from adult *Hyp* mice (Fig. 2C), we speculated that the levels of extracellular Pi might influence the expression of *Slc20a1* on a long-term basis (longer than 24 hours). To test this idea, we used a murine osteoblastic cell line MC3T3-E1, since the primary osteocytic cells could not be maintained in culture for a long time. We cultured MC3T3-E1 cells in the presence of 0.5 mM or 4 mM Pi for 14 days and analyzed the expression of *Slc20a1.* The *Slc20a1* expression was significantly weaker in the cells cultured in the presence of 4 mM Pi than in those cultured in the presence of 0.5 mM Pi (Fig. 6), suggesting that Pi availability in the microenvironment might regulate the *Slc20a1* expression in the cells of osteoblast/osteocyte lineage.

### Direct Effects of 1,25(OH)_2_D_3_ on Gene Expression in the Isolated Osteoblasts and Osteocytes of WT and *Hyp* Mice

**Figure 7:**
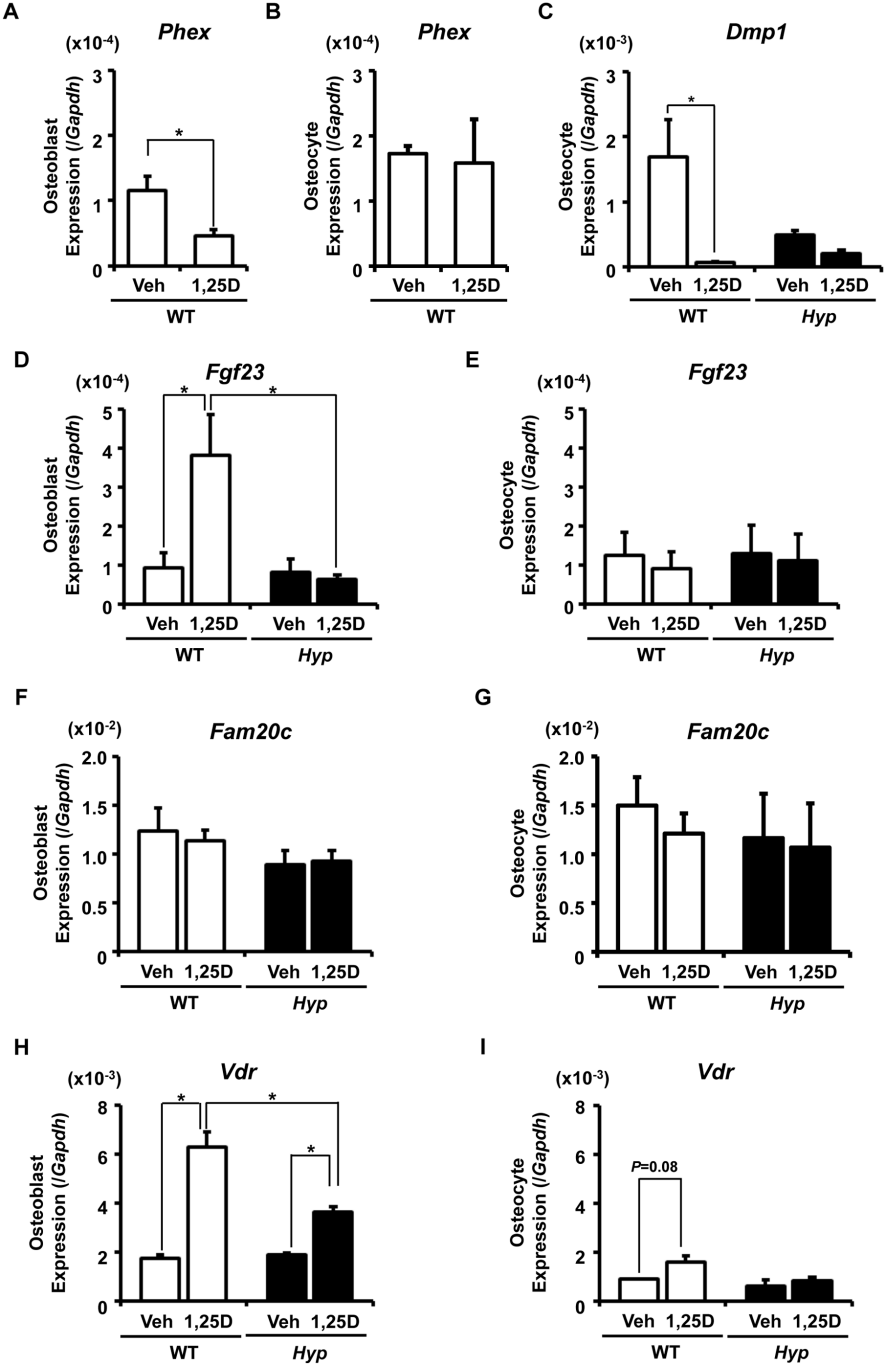
Direct effects of 1,25(OH)_2_D_3_ on gene expression in WT and *Hyp* osteoblastic and osteocytic cells. The cells of osteoblast-rich Fractions 3–5 (**A, D, F, H**) or osteocyte-rich Fractions 6–9 (**B, C, E, G, I**) isolated from 10-week-old WT (white bars) and *Hyp* (black bars) bones were embedded in collagen gel and incubated in the presence of 10^−8^ M 1,25(OH)_2_D_3_ or vehicle (0.1% ethanol) for 24 hours, and the expression of the indicated genes was analyzed by real-time PCR. Expression of the target cDNA was standardized to that of *Gapdh*. Data are shown as the mean ± SEM of 3 isolations. In each isolation procedure, 4 mice were used. *p<0.05.

The effects of 1,25(OH)_2_D_3_ on isolated osteoblastic and osteocytic cells were also examined (Fig. 7). Cells of osteoblast-rich Fractions 3–5 and osteocyte-rich Fractions 6–9 from WT and *Hyp* mice were subjected to collagen-embedded culture and were treated with 10^−8^ M 1,25(OH)_2_D_3_ or vehicle (0.1% ethanol) for 24 hours. The *Phex* expression was decreased by the 24-hour treatment with 1,25(OH)_2_D_3_ in WT osteoblastic cells but not in osteocytic cells (Fig. 7A, B). Interestingly, treatment with 1,25(OH)_2_D_3_ resulted in a marked decrease in *Dmp1* expression in WT osteocytic cells (Fig. 7C). Similarly to the case of Fig. 5C, the expression of *Dmp1* was not higher in *Hyp* cells after the culture. The expression of *Fgf23* in osteoblastic cells was markedly increased by the 24-hour treatment with 1,25(OH)_2_D_3_ in WT, but not in *Hyp* (Fig. 7D). On the other hand, the *Fgf23* expression in osteocytic cells was not altered by the 24-hour treatment with 1,25(OH)_2_D_3_ in either WT or *Hyp* (Fig. 7E). The expression of *Fam20c* in osteoblastic and osteocytic cells was unaltered by the treatment with 1,25(OH)_2_D_3_ in both genotypes (Fig. 7F, G). We also analyzed the expression of *vitamin D receptor* (*Vdr*), which is known to be a target of 1,25(OH)_2_D_3_ in some cell types [30], [31]. Its expression in osteoblastic cells was significantly increased by the treatment with 1,25(OH)_2_D_3_ in both genotypes, although the increase was smaller in *Hyp* (Fig. 7H). On the other hand, the osteocytic expression of *Vdr* was not significantly increased by the treatment with 1,25(OH)_2_D_3_ in either genotype (Fig. 7I).

### Increased Expression of the Genes for FGF Ligands and their Receptors in *Hyp* Osteocytic Cells

**Figure 8:**
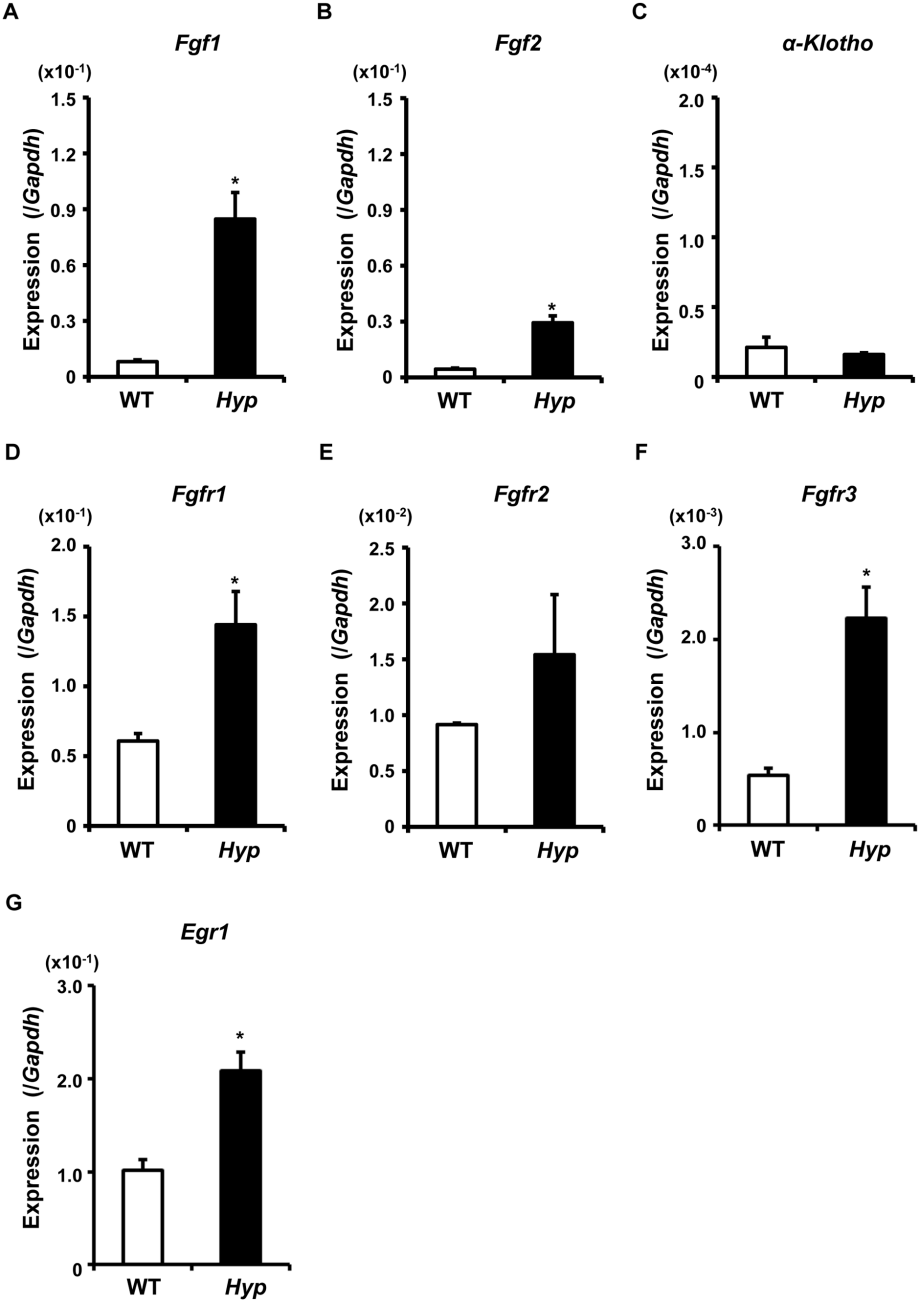
Increased expression of *Fgf1*, *Fgf2*, *Fgfrs* and *Egr-1* in *Hyp* osteocytic cells. RNA was extracted from the osteocytes-rich Fractions 6–9 freshly isolated from 10-week-old WT (white bars) and *Hyp* (black bars) bones, and real-time PCR was performed to examine the expression of the indicate genes. Data are shown as the mean ± SEM of 3 isolations. In each isolation procedure, 4 mice were used. *p<0.05 vs. WT.

It has been previously suggested that the up-regulation of FGF23 in *Hyp* bone might be attributed to the activation of FGFR signaling [32]. Therefore, we examined the expression of the genes encoding FGF1, FGF2, FGFR1–3, and alpha-Klotho in the fresh cells of osteocyte-rich Fractions 6–9 isolated from WT and *Hyp* long bones (Fig. 8). The expression of *Fgf1*, *Fgf2*, *Fgfr1,* and *Fgfr3* was significantly increased in *Hyp* cells, while that of *alpha-Klotho* was low in both genotypes. The expression of *Egr-1*, a target gene of FGF/FGFR signaling, was also found to be up-regulated in *Hyp* osteocytic cells, suggesting the activation of the signaling (Fig. 8).

## Discussion

Accumulating evidence provided by both human diseases and mouse models suggests the profound role of osteocytes in mineral metabolism. FGF23, a central regulator of Pi and vitamin D metabolism, is mainly produced by osteocytes [10], [17], [23]. In addition, some molecules responsible for hereditary hypophosphatemic rickets, such as PHEX and DMP1, are also highly expressed in osteocytes [16], [17], [18]. The inactivation of *PHEX/Phex* or *DMP1/Dmp1* has been shown to result in the increased expression of *FGF23/Fgf23*, leading to increased renal Pi wasting and hypophosphatemia in both humans and mice [17], [18], [22], [23]. *FAM20C/Fam20c,* which is expressed in mineralized tissue including osteocytes, has also been shown to be involved in Pi metabolism. *Fam20c*-deficient mice exhibit hypophosphatemia associated with the increased levels of *Fgf23,* and a loss-of-function mutation in the gene has been identified in patients who manifested hypophosphatemia [19], [21]. These findings suggest that the multiple osteocytic genes functionally interact with each other to regulate mineral metabolism.

We here attempted to clarify the complex influence of inactivation of the *Phex* gene on the expression of the various osteocytic genes involved in mineral metabolism using *Hyp* mice, a model for human XLH. To obtain detailed gene expression profiles in osteoblast/osteocyte lineage cells based on the differentiation stages, we used cells freshly isolated from the long bones of *Hyp* and WT mice by sequential digestion with collagenase and decalcification with EGTA. The expression profiles of the osteoblastic marker *Kera* and osteocytic marker *Sost* in fresh cells from each fraction suggested that Fractions 6/7 and 8/9 were osteocyte-rich in both WT and *Hyp*, while the earlier fractions were osteoblast-rich (Fig. 1, 2B). Stern et al. also recently demonstrated that the combination of collagenase digestion and EDTA-based decalcification enabled the isolation of osteocytes from the long bones of mature young (4-month-old) and aged (22-month-old) mice [33]. In our experiments, we found that *Phex, Fgf23, Dmp1*, and *Fam20c* were all more highly expressed in the osteocyte-rich later fractions than in the osteoblast-rich earlier fractions (Fig. 1C, 2C), which enhanced the critical roles of osteocytes in mineral metabolism.

The impaired renal Pi reabsorption and decreased 1,25(OH)_2_D production in human XLH and *Hyp* mice have been attributed to the increased levels of FGF23 [22], [23]. We confirmed that *Fgf23* mRNA levels were increased in osteocytes isolated from 20-week-old *Hyp* mice. Unexpectedly, the *Fgf23* mRNA level was increased in *Hyp* cells in Fr. 6/7 but not in Fr. 8/9, suggesting that the increase was more evident in younger osteocytes that just had been embedded in bone matrix than in mature osteocytes. In addition, this increase in *Fgf23* mRNA was smaller than expected from the serum level (Fig. 2A, C). Based on these results, we speculate that circulating levels of FGF23 were likely to be regulated at the posttranslational level as well as at the mRNA level. This is consistent with a previous report by Yuan, et al., which demonstrated that the degradation of FGF23 as well as its production was regulated in a Phex-dependent manner [34]. Since *O*-glycosylation has been shown to be involved in completing the processing of intact FGF23, we examined the expression of *Galnt3* encoding GalNAc-transferase 3, which is responsible for *O*-glycosylation of FGF23. Although Liu, et al. reported the reduced expression of *Galnt3* in *Hyp* cortical bones based on a gene array analysis [35], we could not detect significant differences in *Galnt3* expression between the genotypes. Interestingly, both serum FGF23 level and *Fgf23* mRNA in bones were markedly higher in *Hyp* fetuses than in WT fetuses in spite of similar serum Pi levels, and this elevation was more pronounced than that at 4 weeks of age (Fig. 3). Consistent with this observation, the expression of *Fgf23* was previously shown to be suppressed in juvenile *Hyp* bones when explanted into adult *Hyp* mice, which suggested the presence of an age-dependent systemic inhibitor of *Fgf23* expression [36].

The expression of *Dmp1* and *Fam20c* was markedly increased in osteoblast/osteocyte lineage cells isolated from *Hyp* mice, and their elevated expression was observed in earlier fractions as well as later fractions (Fig. 2C). The immunostaining confirmed the increased Dmp1 expression in *Hyp* osteocytes (Fig. 2D). The increased expression of *Dmp1* and *Fam20c* in *Hyp* bones emerged before birth when serum Pi levels were similar between the genotypes, as did that of *Fgf23* (Fig. 3). These results suggested that inactivation of the *Phex* gene in *Hyp* mice led to the increased expression of *Dmp1* and *Fam20c* as well as that of *Fgf23*. The expression of both FGF23 and DMP1 was previously shown to increase in the bones of patients in the early stages of chronic kidney disease (CKD) [37]. These observations as well as our findings shown here suggest that FGF23 and DMP1 can be up-regulated together in some conditions.

Na^+^/Pi co-transporters in mammals have been classified into 3 groups; type I, II and III [38]. Among them, type I co-transporters transport organic anions rather than Pi. Type IIa and IIc co-transporters are predominantly expressed in the renal proximal tubules, while type IIb functions in the intestine and placenta. Type III co-transporters Pit1 and Pit2 were shown to be expressed ubiquitously [38]. In this study, we found that the expression of *Slc20a1* encoding Pit1 was higher in osteoblasts and osteocytes freshly isolated from hypophosphatemic adult *Hyp* mice than in those from WT mice (Fig. 2C). On the other hand, no significant difference was observed in *Slc20a1* expression in fetal bones between *Hyp* and WT at E18.5, in which serum Pi levels were similar potentially due to increased materno-fetal Pi transport via the placenta (Fig. 3). We speculate that increased *Slc20a1* expression in the osteoblasts and osteocytes from adult *Hyp* mice may be a compensatory mechanism to adapt to the postnatal decrease in extracellular Pi concentrations. Consistent with this notion, we found that the expression of *Slc20a1* was stronger in osteoblastic MC3T3-E1 cells cultured for 14 days in the presence of 0.5 mM Pi than in the cells cultured in the presence of 4 mM Pi (Fig. 6). However, the 24-hour treatment with high Pi did not alter the expression of *Slc20a1* in isolated osteoblasts and osteocytes (Fig. 5H, I). It may take some time before the low availability of Pi leads to the increased expression of *Slc20a1*. Since the increase in *Slc20a1* expression in *Hyp* cells was evident in Fractions 4 to 8/9, but not in Fr. 3, the requirement for Pi may be higher in mature osteoblasts and osteocytes than in early osteoblasts (Fig. 2C).

FGF23 produced in bone exerts its effects on distant organs including the kidney in an endocrine fashion. Reflecting its endocrine function, the production of FGF23 has been suggested to be regulated by various systemic factors as well as local factors [39]. In this study, we performed collagen-embedded culture of isolated primary osteoblasts and osteocytes to examine the effects of Pi and 1,25(OH)_2_D_3_ on the gene expression. When cultured in type I collagen gel, the cells of Fractions 6–9 from WT mice retained the expression of the osteocytic genes *Sost* and *Phex* for 48 hours, and the expression of the osteoblastic marker *Kera* remained low (Fig. 4), suggesting that the cells kept their osteocytic phenotype to some extent during the culture. However, the expression of *Dmp1*, *Fgf23* and *Fam20c* in fresh *Hyp* osteocytic cells was not higher than in WT cells after 24-hour culture, although the *Slc20a1* expression was still higher in *Hyp* osteocytic cells than in WT cells after the culture (Fig. 5, 7). Once taken out from bone, *Hyp* cells might lose the expression of some genes more quickly than WT cells by some unidentified mechanism.

Pi is one of the systemic factors that may influence the circulating level of FGF23. Pi loading was shown to increase FGF23 levels in mice, although it remains unclear whether dietary Pi regulates FGF23 levels in humans [40], [41], [42]. Considering these observations, it is reasonable to hypothesize that extracellular Pi itself exerts its effects on osteocytes to regulate gene expression. Several studies including ours previously reported that an increase in the level of extracellular Pi itself triggered signal transduction in some skeletal cell types such as osteoblasts and chondrocytes. Beck et al. demonstrated that extracellular Pi itself functioned as a signaling molecule in the osteoblastic cell line MC3T3-E1 to regulate the expression of several genes including that for osteopontin [43], [44]. We previously reported that the treatments with a higher concentration of extracellular Pi induced the up-regulation of *cyclin D1* to facilitate the proliferation of early chondrocytes [45]. As described above, the postnatal increase in *Slc20a1* expression in *Hyp* osteoblasts and osteocytes shown here suggests that osteoblasts and osteocytes may adapt to chronic low Pi availability. In addition, we found that the 24-hour treatment with an elevated concentration of Pi resulted in the increased expression of *Dmp1* in WT osteocytic cells (Fig. 5C), which indicated that the osteocytes responded to an alteration in the level of extracellular Pi. However, the expression of *Fgf23* in osteoblasts and osteocytes was not altered in either WT or *Hyp* by the 24-hour treatment with high extracellular Pi (Fig. 5D, E). A previous study reported that acute changes in serum Pi did not influence FGF23 levels in healthy humans [46]. We cannot exclude the possibility that longer treatment with Pi may have altered the expression of *Fgf23*, *Fam20c*, or *Phex.* It is possible that chronic exposure to increased extracellular Pi may facilitate the differentiation of osteoblasts to osteocytes by inducing the expression of *Dmp1*, which may be associated with elevated expression of *Fgf23*.

We also examined the acute effects of 1,25(OH)_2_D_3_ on isolated primary osteoblasts and osteocytes. It is well known that the expression of *Vdr* is up-regulated by 1,25(OH)_2_D_3_ in some cell types [30], [31]. In the current study, the 24-hour treatment with 1,25(OH)_2_D_3_ resulted in the increased expression of *Vdr* in the collagen-embedded culture of osteoblastic cells in both WT and *Hyp*, confirming the responsiveness of osteoblasts to 1,25(OH)_2_D_3_ (Fig. 7H). Since the effects of 1,25(OH)_2_D_3_ on the *Vdr* expression in osteocytic cells were not significant in both genotypes, the response to 1,25(OH)_2_D_3_ might differ between osteoblasts and osteocytes (Fig. 7I). As to *Fgf23*, previous reports demonstrated that 1,25(OH)_2_D_3_ increased its expression in rat osteoblastic cell lines [47]. Here we found that the 24-hour treatment with 1,25(OH)_2_D_3_ increased the expression of *Fgf23* in osteoblastic cells but not in osteocytic cells from WT mice (Fig. 7D, E), suggesting that the effects of 1,25(OH)_2_D_3_ on the *Fgf23* expression might depend on the differentiation stage of the cells. Ito, et al. recently examined the effects of Pi and 1,25(OH)_2_D_3_ on gene expression on a newly established osteocyte-like cell line IDG-SW3, and reported that treatment with 10 nM 1,25(OH)_2_D_3_ up-regulated the expression of *Fgf23* in the presence of 1 mM Pi, but not in the presence of 4 or 10 mM Pi [48]. These results suggest that the effects of 1,25(OH)_2_D_3_ on *Fgf23* expression may be context dependent and be influenced by other factors such as the differentiation stage of the cells and the concentration of extracellular Pi. Because Dmp1 negatively regulates the expression of *Fgf23* [17], [18], a marked reduction in the expression of *Dmp1* by 1,25(OH)_2_D_3_ may be followed by an increase in *Fgf23* expression in osteocytes.

In *Hyp*, the 24 hour-treatment with 1,25(OH)_2_D_3_ failed to induce the expression of *Fgf23* even in osteoblastic cells (Fig. 7D). In addition, the increase in the *Vdr* expression by the treatment with 1,25(OH)_2_D_3_ was smaller in *Hyp* osteoblasts than in WT osteoblasts (Fig. 7H). These results suggest that the responsiveness to 1,25(OH)_2_D_3_ might be reduced in *Hyp* cells.

Induction of FGF23 in *Hyp* bone has previously been attributed to the activation of FGFR signaling [32]. In addition, activating mutations in human FGFR1 cause osteoglophonic dysplasia (OMIM #166250) associated with increased levels of FGF23 and hypophosphatemia [49], which also suggests that activated FGF/FGFR signaling may play a role in regulation of *Fgf23* expression. Therefore, we here examined the expression of *Fgf1*, *Fgf2*, and *Fgfr1–3* in the fresh osteocytic cells isolated from WT and *Hyp* bones. It was interesting the expression of multiple FGF ligands and their receptors was significantly up-regulated in *Hyp* cells (Fig. 8). A previous report also demonstrated the increased expression of *Fgfr1* in *Hyp* bone, although the expression of the genes for other FGF receptors and ligands was not examined [50]. These results implicate the activation of FGF/FGFR signaling in *Hyp* osteocytic cells. Indeed, the expression of *Egr-1*, which is a target of FGF/FGFR signaling, was clearly increased in *Hyp* osteocytic cells (Fig. 8G). The activation of FGF/FGFR signaling in osteocytes might play roles in the pathogenesis of *Hyp* mice. It was previously reported that treatment with FGF2 induced the expression of *Dmp1* in a murine osteocytic cell line MLO-Y4 [51]. Considering their results together with ours, the enhanced FGF/FGFR signaling might be responsible for the increased expression of *Dmp1* in *Hyp* osteocytes.

In conclusion, we found that the inactivation of *Phex* in *Hyp* mice resulted in the increased expression of *Dmp1*, *Fam20c,* and *Slc20a1,* as well as *Fgf23* in osteocytes. The up-regulation of *Dmp1*, *Fam20c*, and *Fgf23* in *Hyp* bone occurred in the fetal stage. Activated FGF/FGFR signaling in *Hyp* osteocytic cells was suggested by the increased expression of the genes for multiple FGF ligands, their receptors, and Egr-1, which might contribute to the pathogenesis in *Hyp* mice. The method we used for isolation of osteoblast/osteocyte lineage cells might be useful to analyze differential gene expression and function between osteoblasts and osteocytes.

## Supporting Information

Checklist S1 Completed ARRIVE (Animal Research: Reporting of *In Vivo* Experiments) Guidelines Checklist.
(PDF)
